# A CSRR-Fed SIW Cavity-Backed Fractal Patch Antenna for Wireless Energy Harvesting and Communication

**DOI:** 10.3390/s150921196

**Published:** 2015-08-28

**Authors:** Hailin Cao, Fen Jiang, Jiujiu Liu, Wenbin Cai, Mingchun Tang, Xiaoheng Tan, Shizhong Yang

**Affiliations:** 1 State Key Laboratory of Power Transmission Equipment & System Security and New Technology, Chongqing University, Chongqing 400000, China; E-Mail: ysz@cqu.edu.cn; 2 Key Laboratory of Aerocraft Tracking, Telemetering & Command and Communication (Ministry of Education), Chongqing University, Chongqing 400000, China; E-Mails: fenjiang2617@gmail.com (F.J.); liujiujiu36@163.com (J.L.); caiwenbinsky@163.com (W.C.); 3 College of Communication, Chongqing University, Chongqing 400000, China; E-Mails: tangmingchun@cqu.edu.cn (M.T.); txh@cqu.edu.cn (X.T.)

**Keywords:** substrate-integrated waveguide (SIW), dual-band, dual-polarized, complementary split-ring resonator (CSRR), patch antenna

## Abstract

This paper presents a novel compact dual-band and dual-polarized complementary split-ring resonator (CSRR)-fed substrate-integrated waveguide (SIW) cavity-backed fractal patch antenna for wireless energy harvesting and communication. The proposed antenna is composed of a Giuseppe Peano fractal radiation patch with a backed SIW cavity. To enhance the bandwidth and minimize the dimensions, the CSRR structure is designed to feed the Giuseppe Peano fractal patch orthogonally. A prototype of the proposed antenna is simulated, fabricated and measured. The proposed antenna exhibits good directionality and high cross-polarization level with especially compact size.

## 1. Introduction

Microwave power transmission (MPT) transmits power through free-space without the use of wires or cables, which is attractive for applications in oil-less helicopters, 24 h high-altitude platforms and solar power satellites. Recently, MPT technology has also been used to supply power for wireless sensors, radio frequency identification, and portable electrical devices. The power amplifiers for MPT often work in the nonlinear area, but the wireless communication transmitter generally works in the linear area to obtain a lower bit error rate (BER). Thus, a dual-band antenna that can be applied simultaneously for wireless energy harvesting and communication with compact size is highly desirable.

The emerging substrate integrated waveguide (SIW) technique has gained renewed attention for the integration of planar and non-planar circuits into a simple and low-cost PCB or LTCC fabrication process [1,2]. Then, the traditional waveguide, which is costly with a bulky non-planar hollow rectangular structure and high power capacity, can be synthesized into a planar substrate with metalized slots or even arrays of metallic via posts. The SIW has provided very attractive applications in designing low-cost, low-profile, and highly efficient waveguide microwave printed circuitry, active devices, and antennas. Benefitting from superior characteristics, which include significant surface wave suppression, reduced coupling, better matching, and wider scanning, SIW cavity-backed patch antennas have been extensively studied and employed [3,4,5,6,7,8,9]. In [3,4,5], various cavity-backed antennas with different cavity shapes and patch structures were discussed. In [6], SIW cavity-backed dual-band circularly polarized (CP) antennas with planar configurations were investigated. A dual-mode SIW cavity-backed triangular ring slot antenna was proposed and developed for dual-band application in [8]. To increase the bandwidth, an SIW-fed cavity-backed rectangular patch antenna was proposed in [9], and it was designed to operate in its TE210 mode. However, with the demand for compact wireless terminals, designing a planar antenna that occupies minimal circuit board area is still a challenge. The use of metamaterials has been proven to be an efficient approach for reducing the antenna size while maintaining satisfactory antenna performance. In [10], a miniaturized magnetic dipole antenna is proposed, which is based on the mechanism of the conventional SRR. The fractal geometries are also conducive to the miniaturization of antenna designs and realization of antenna ultra-wideband or multiband characteristics [11,12].

Alternately, dual-band and dual-polarized antennas have attracted much attention with the increasing demand for polarization diversity in dual-band wireless communication [13,14]. However, dual-polarized microstrip antennas are generally associated with narrow frequency bandwidth and poor isolation performance [13]. To explore a greater operational frequency range, a dual-band and dual-polarized SIW fed slot antenna was investigated in [14], which is constructed with the usage of a frequency selective surface right above it. 

This paper presents a novel compact dual-band and dual-polarized CSRR-fed SIW cavity-backed fractal patch antenna, which aims to work simultaneously for wireless energy harvesting and communication. The proposed antenna utilizes an SIW cavity. To reduce the dimension, a Giuseppe Peano fractal patch has been employed. For further performance enhancement, a CSRR-fed network has been utilized to substantially lower the resonant frequency. The proposed antenna has been analyzed, fabricated and tested.

## 2. Design Section

### 2.1. Antenna Configuration and Design Consideration

**Figure 1:**
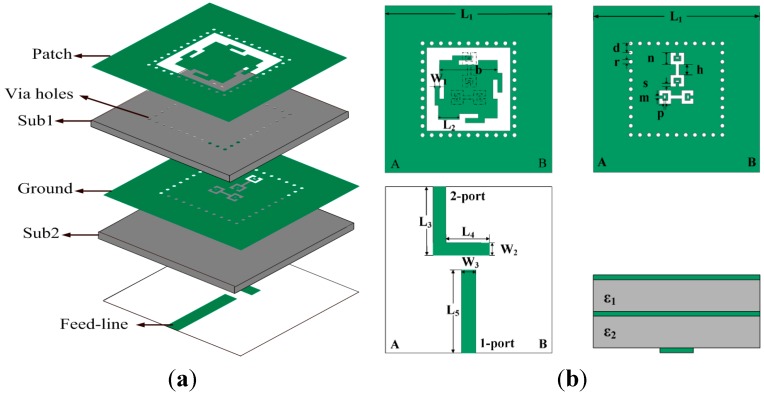
Configuration of the proposed antenna: (**a**) 3-dimensional view; and (**b**) top and side view of each metallic layer. (*L*_1_ = 40 mm, *L*_2_ = 4.6 mm, *L*_3_ = 16.5 mm, *L*_4_ = 11.5 mm, *L*_5_ = 20 mm. *W*_1_ = 1.1 mm, *W*_2_ = 3 mm, *W*_3_ = 3.4 mm, *b* = 13.9 mm, *d* = 2.2 mm, *r* = 0.3 mm, *n* = 2.9 mm, *h* = 2.5 mm, *s* = 0.8 mm, *m* = 0.7 mm, *p* = 0.4 mm).

The configuration of the proposed CSRR-fed SIW cavity-backed fractal patch antenna is illustrated in Figure 1. It consists of three metallization layers and a stack of two substrates with thicknesses of *h*_1_ and *h*_2_ and relative dielectric constants of *ε_r_*_1_ and *ε_r_*_2_, respectively. Many via holes spaced along the rectangular opening are drilled in the substrate，thus constituting SIW cavity, which is the backing of the radiating element (patch surrounded by a slot). A Giuseppe Peano fractal geometry radiation patch is printed on the top metallization layer, which is fed by two longitudinal and transverse CSRR slots etched on the middle metallization layer. The microstrip feed lines are on the bottom layer of the lower substrate. The proposed antenna is printed on two substrates with the same size, 40 × 40 mm^2^. Two layers have the same substrate material, F4B, with the relative dielectric constant of *ε_r_*_1_ = *ε_r_*_2_ = 2.65, the thickness of *h*_1_ = *h*_2_ = 0.8 mm, and the loss tangent of *tanδ* = 0.002. All vias have the same diameter of 0.3 mm, and the spacing between each is 2.2 mm [3]. The detailed geometrical parameters are illustrated in Figure 1.

### 2.2. Design Evolution

This sub-section presents the design procedure of the proposed antenna in detail. The geometrical parameters are illustrated in Figure 1. To minimize the mutual coupling between the feed lines and the patch, the feed lines are printed on the bottom layer of the antenna. Two orthogonal feed ports, *i.e.*, 1-port and 2-port, excite vertical and horizontal polarized waves, respectively. The CSRR slots are placed in a “T” orthogonal configuration under the fractal patch to improve the isolation between the two feed ports. The CSRR structure (which is based on the metamaterial concept) [9,10] and Giuseppe Peano fractal geometry have been adopted to minimize the dimensions of the antenna.

According our design method, an antenna with good radiation performance would be obtained in terms of the realized gain and the compact dimensions.

#### 2.2.1. The Influence of the Coupling Aperture

Compared with classical edge-fed or probe-fed microstrip antennas, the slot-coupled microstrip antenna has many advantages, such as easy integration into arrays with active circuits. In [13], an H-shaped slot is used to couple a microstrip antenna for a wider bandwidth and easy integration with active circuits. The use of metamaterials has been proven to be an efficient approach to reducing the antenna size while maintaining satisfactory antenna performance [10].

**Figure 2:**
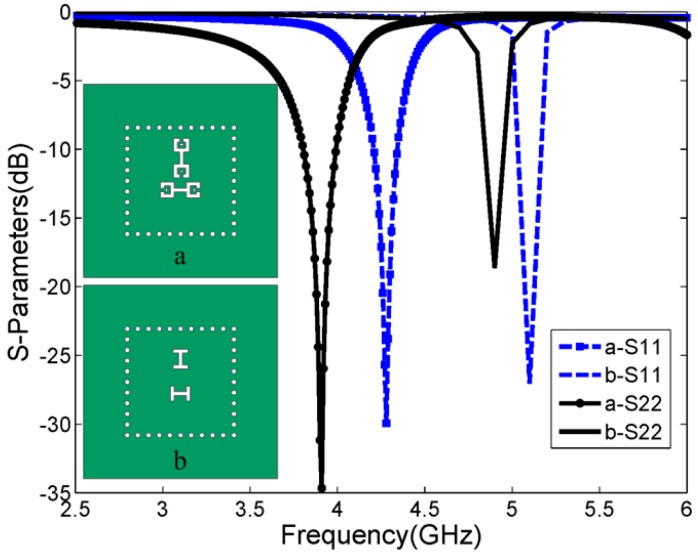
Simulated return loss with different coupling structures.

In the process of antenna optimization, a CSRR slot based on the metamaterial concept has been adopted to couple the Giuseppe Peano fractal radiation patch. The dimensions of the CSRR shape (a) are the same as those in Figure 1. The H-shaped (b) slot has two 0.8-millimeter-wide and 2.5-millimeter-high apertures with the same width as that of the CSRR. Two coupled structures are located at the same position. The comparative study was carried out using HFSS to discuss the effects of different coupling apertures, as shown in Figure 2. The conventional H-shaped slot-fed antenna has dual-band resonant frequencies at 4.9 GHz and 5.1 GHz. In contrast, the proposed CSRR-fed patch has dual-band resonant frequencies at 3.9 GHz and 4.3 GHz. It can be seen that the fractal patch fed with the proposed CSRR structure achieves a dual-band resonance at much lower resonant frequencies. This novel coupled structure provides an excellent strategy for minimization of the slot-coupled antenna size.

#### 2.2.2. The Influence of the Radiation Patch Shape

Fractal geometry can be used to miniaturize patch antennas by increasing the effective electrical length of the radiator in the limited area. Consequently, fractal structures can be applied to the compact antenna designs, whose radiation patterns and return loss characteristics are similar to those of larger antennas [11,12].

**Figure 3:**
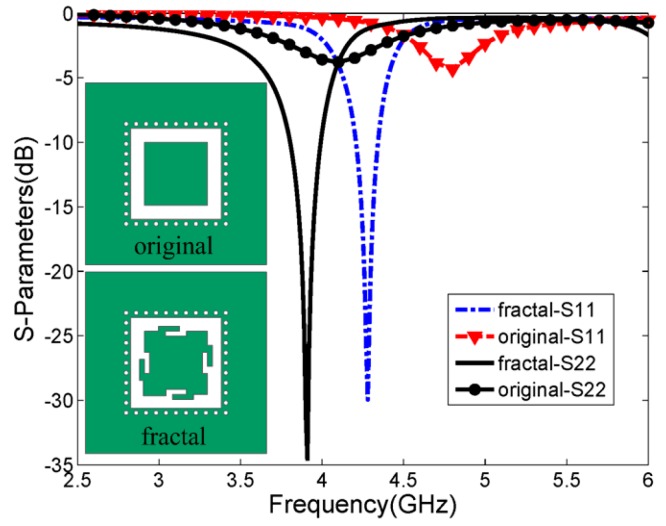
Simulated return loss with different radiator shapes.

The Giuseppe Peano fractal geometry is applied to the radiation patch in this design. The HFSS software was also used for a comparative numerical study between the original rectangular and the proposed fractal geometry radiators, which were both coupled with the CSRR slot. The dimensions of the Giuseppe Peano fractal are the same as those illustrated in Figure 1, and the original radiator patch is in a square shape of 13.9 mm × 13.9 mm. The return loss versus frequency curves of the antenna are illustrated in Figure 3. As shown in Figure 3, the original square radiator resonated at both 4.1 GHz and 4.8 GHz. In comparison, the proposed Giuseppe Peano fractal radiator resonates at both 3.9 GHz and 4.3 GHz. With the Giuseppe Peano fractal geometry, the current of the proposed antenna should be forced to travel along the convoluted path instead of a straight Euclidean path. It can be seen that the decrease of the resonant frequencies of the fractal patch antenna is quite obvious. Furthermore, the fractal antenna has significant improvement of the return loss level at its operating frequencies. It should be noted that the bandwidth at the resonant frequency of the Giuseppe Peano fractal patch is slightly narrower than that of the square patch.

## 3. Results and Discussion

**Figure 4:**
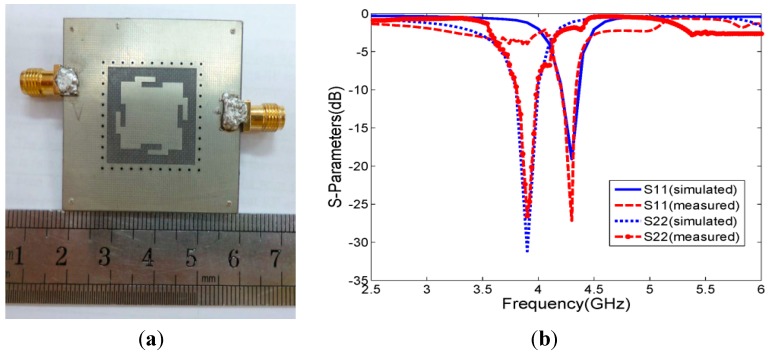
(**a**) Photograph of the proposed antenna; (**b**) Simulated and measured S11 and S22 of the proposed antenna.

A photo of the fabricated antenna is illustrated in Figure 4a. The measured and simulated results of return loss in terms of S11 of the 1-port and S22 of the 2-port are displayed in Figure 4b. For the two polarizations, the measured values of S11 are −30 dB and −34 dB at their corresponding center frequencies.

The return loss achieves a bandwidth of 3.3% from 4.2 to 4.34 GHz (for a 10-dB return loss) at 1-port and a bandwidth of 5.1% from 3.8 to 4.0 GHz at 2-port. All the data are measured with an Agilent E8362C Vector Network Analyzer (VNA). A reasonably good agreement between the simulation results and measured results is observed.

**Figure 5:**
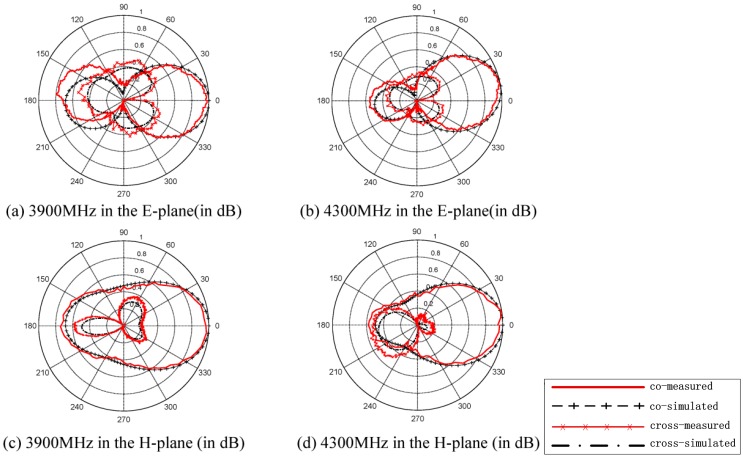
Measured and simulated co- and cross-polarizations of the proposed antenna in the E-plane of (**a**) 3800 MHz and (**b**) 4300 MHz and the H-plane of t (**c**) 3900 MHz and (**d**) 4300 MHz.

The far field radiation patterns of the proposed antenna are shown in Figure 5, which are obtained from HFSS and tested in an anechoic chamber. The simulated and measured E-plane and H-plane far field radiation patterns of 1-port at 3.9 GHz are presented in Figure 5a,c, respectively. The cross-polarization components are also illustrated. In this case, higher cross-polarization levels are observed at the E-plane than at the H-plane. The cross-polarization levels are approximately 24-dB and 22-dB lower than the co-polarization component at the boresight in the E- and H-planes, respectively, indicating excellent polarization purity. Figure 5b,d present the measured and calculated radiation patterns in the E- and H-planes of the 2-port, respectively. The cross-polarization components are approximately 24-dB and 21-dB lower than the co-polarization component at the boresight in the E- and H-planes. The measured gain of the fabricated antenna is 4.6 dBi at 3.9 GHz and 5.9 dBi at 4.3 GHz, respectively. In general, the calculated co-polarization patterns agree well with the measured ones.

## 4. Conclusions

In this paper, a novel compact dual-band and dual-polarized CSRR-fed SIW cavity-backed fractal patch antenna, which aims to work simultaneously for wireless energy harvesting and communication, is proposed and analyzed. It is shown that the combination of fractal geometries and the CSRR feed is effective in the design of compact SIW antennas. A prototype of the proposed antenna is simulated, fabricated and measured. The tendencies of the measured results are in agreement with those of the simulation. The proposed antenna exhibits good gains and high cross-polarization levels, especially for the miniaturizations. In addition to the inherent advantages of significant surface wave suppression, excellent impedance matching, and a wider scan range, the proposed antenna has the advantages of low cost and good integration ability, which make it suitable for dual-band and dual-polarized applications and future wireless energy harvesting and communication array applications. 
